# Supplementary material for: Evidence of Virulent Multi-Drug Resistant and Biofilm-Forming Listeria Species Isolated from Various Sources in South Africa
Source: Pathogens. 2022 Jul 27;11(8):843. doi: 10.3390/pathogens11080843 (PMC9416180; doi:10.3390/pathogens11080843)
Supplement: Supplementary file 1 [file pathogens-11-00843-s001.zip › pathogens-1787560-supplementary.pdf]

Table S1. List of oligonucleotide primer sequences and PCR conditions used for the identification of *Listeria* spp.

Primers	Sequences (5′-3′)	Target Genes	Amplicon size (bp)	Annealing T°c	PCR Conditions	References
27F<br>1492R	AGAGTTTGATCCTGGCTCAG<br>GGTTACCTTGTTACGACTT	*16S rRNA*	1420	59	94 °C for 10 min, 25 cycles, 97 °C for 1 min, annealing temperature for 1 min and 72 °C for 1 min 30 s, and final extension at 72 °C for 15 min.	[1]
PRS F<br>PRS R	GCTGAAGAGATTGCGAAAGAAG<br>CAAAGAAACCTTGGATTTGCGG	*prs*	370	54	5 min at 94 °C, 35 cycles, 94 °C for 45 s, annealing temperature for 30 s and 72 °C for 1 min, and a final extension of 72 °C for 10 min.	[2]
PRFA F<br>PRFA R	GAT ACA GAA ACA TCG GTT GGC<br>GTGTAA TCT TGA TGC CAT CAG	*prfA*	274	56	94 °C for 5 min, 33 cycles, 94 °C for 45 s, annealing temperature for 30 s and extension at 72 °C for 1 min, and final extension at 72 °C for 5 min.	[2]
JOGRAYIF<br>JOGRAYIR	GCGGATAAAGGTGTTCGGGTCAA<br>ATTTGCTATCGTCCGAGGCTAGG	*oxidoreductase*	201	58	94 °C for 5 min, 35 cycles, annealing temperature for 30 s and 72 °C for 30 s, final extension 72 °C for 5 min.	[3]
LIN0464F<br>LIN0464R	CGCATTTATCGCCAAAACTC<br>TCGTGACATAGACGCGATTG	*lin0464*	749			
LIV22228F<br>LIV22228R	CGAATTCCTTATTCACTTGAGC<br>GGTGCTGCGAACTTAACTCA	*namA*	463			
LMO1030F<br>LMO1030R	GCTTGTATTCACTTGGATTTGTCTGG<br>ACCATCCGCATTCTCAGCCAACT	*lmo1030*	509			
ISEELINF<br>ISEELINR	GTACCTGCTGGGAGTACATA<br>CTGTCTCCATATCCGTACAG	*lmo0333*	673			
LWE1801F<br>LWE1801R	CGTGGCACAATAGCAATCTG<br>GACATGCCTGCTGAACTAGA	*scrA*	281			

Table S2. List of oligonucleotide primer sequences and PCR conditions used for detection of virulence genes in *L. monocytogenes*.

Primers	Sequences (5′-3′)	Target Genes	Amplicon Size (bp)	Annealing T °C	PCR Conditions	References
inlAF	CCTAGCAGGTCTAACCGCAC	*inlA*	256	52	94 °C for 5 min, 35 cycles, 94 °C for 35 s, annealing temperature for 30 s and 72 °C for 1 min, and a final extension for 10 min at 72 °C.	[4]
INLAR	TCGCTAATTTGGTTATGCCC					
INLBF	TGATGTTGATGGAACGGTAAT	*inlB*	272	52		[5]
INLBR	CTCGTGGAAGTTTGTAGATGC					
INLCR	AATTCCCACAGGACACAACC	*inlC*	517	52		[6]
INLCF	CGGGAATGCAATTTTTCACTA					
INLJR	TGTAACCCCGCTTACACAGTT	*inlJ*	238	52		
INLJF	AGCGGCTTGGCAGTCTAATA					
ACTAF	CCAAGCGAGGTAAATACGGGA	*actA*	650	52		[7]
ACTAR	GTCCGAAGCATTTACCTCTTC					
HLYAF	ATCATCGACGGCAACCTCGGAGAC	*hlyA*	404	52		[5]
HLYAR	CACCATTCCCAAGCTAAACCAGTGC					
PLCAF	CTCGGACCATTGTAGTCATCTT	*plcA*	326	52		[7]
PLCAR	CACTTTCAGGCGTATTAGAAACGA					
PLCBF	CTGCTTGAGCGTTCATGTCTCATC	*plcB*	289	52		[5]
PLCBR	ATGGGTTTCACTCTCCTTCTAC					
IAPF	ACAAGCTGCACCTGTTGCAG	*iap*	131	52		[8]
IAPR	TGACAGCGTGTGTAGTAGCA					

Table S3. List of oligonucleotide primer sequences and PCR conditions used for detection of antibiotic resistance genes in *L. monocytogenes*.

Primers	Sequen0ces (5′-3′)	Target Genes	Amplicon Size (bp)	Annealing T °C	PCR Conditions	References
erm(A)F	AAGCGGTAAACCCCTCTGAG	*erm(A)*	441	53	95 °C for 4min, 35 cycles, 30 s at 94 °C, annealing temperature for 30 s, 1.5 min at 72 °C, and a final extension step at 72 °C for 7 min.	[9]
erm(A)R	TCAAAGCCTGTCGGAATTGG					
erm(B1)F	CATTTAACGACGAAACTGGC	*erm(B1)*	425	60		
erm(B1)R	GGAACATCTGTGGTATGGCG					
erm(B2)F	GAAAAGGTACTCAACCAAATA	*erm(B2)*	639	58		[10]
erm(B2)F	AGTAACGGTACTTAAATTGTTTAC					
erm(C)F	ATCTTTGAAATCGGCTCAGG	*erm(C)*	295	49		[9]
erm(C)R	CAAACCCGTATTCCACGATT					
erm(T)F	TATTATTGAGATTGGTTCAGGG	*erm(T)*	395	56		
erm(T)R	GGATGAAAGTATTCTCTAGGGATTT					
BlaTEMF	TTC TTG AAG ACG AAA GGG C	*bla*$_{TEM}$	1190	60	94 °C for 60 s at, 30 cycles, 60 s at 94 °C, annealing temperature for 60 s and 72 °C for 60 s, and final extension for 5 min at 72 °C.	[11]
BlaTEMR	ATG GTG AGT GGA ACG AAA AC					

dfr1F	TGGTAGCTATATCGAAGAATGGAGT	*dfr1*	425	52	95 °C for 10 min, 30 cycles, 45 s at 94 °C, annealing temperature for 45 s and 2 min at 72 °C, with a final extension for 10 min at 72 °C.	[12]
dfr1R	TATGTTAGAGGCGAAGTCTTGGGTA					
dfr5F	AGCTACTCTTTAAAGCCTTGACGTA	*dfr5*	341	55		
dfr5R	GTGTTGCTCAAAAACAACTTCG					
dfr12F	GAGCTGAGATATACACTCTGGCACT	*dfr12*	155	60		
dfr12R	GTACGGAATTACAGCTTGAATGGT					
dfr1SF	ATGGAGTGCCAAAGGTGAAC	*dfr1s*	241	52		
dfr1SR	TATCTCCCCACCACCTGAAA					
dfr5SF	TCATTAATGGCTGCAAAAGC	*dfr5s*	460	55		
dfr5sR	CCTTTTGCCAAATTTGATAGC					
dfr7sF	TCTGCAACGTCAGAAAATGG	*dfr7s*	404	55		
dfr7sR	TGCTCAAAAACCAAATTGAAA					
dfr12sF	TTTATCTCGTTGCTGCGATG	*dfr12s*	457	60		
dfr12sR	TAAACGGAGTGGGTGTACGG					
dfr17sF	GAAAATATCATTGATTTCTGCAGTG	*dfr17s*	465	55		
dfr17sR	TTTTTCCAAATCTGGTATGTATAATTT					
pse-1 F	CGCTTCCCGTTAACAAGTAC	*pse-1*	419	57	94 °C for 12 min, 34 cycles, 1 min at 94 °C, annealing temperature for 45 s, 5 min at 72 °C, and 5 min, 5 sat 72 °C for final extension.	[13]
pse-1 R	CTGGTTCATTTCAGATAGCG					
ant F	GTGGATGGCGGCCTGAAGCC	*ant (3″)-la*	526	58	3 min at 94 °C, 35 cycles, 1 min at 94 °C, annealing temperature for 45 s and 1 min at 72° C, and final extension of 5 min at 72 °C.	[14]
ant R	ATTGCCCAGTCGGCAGCG					
tet A F	GCTACATCCTGCTTGCCTTC	*tet A*	210	55	94 °C for 5 min, 34 cycles, annealing temperature for 45 s, 25 s at 94 °C and 50 s at 72 °C, and 5 min at 72 °C for the final extension.	
tet A R	CATAGATCGCCGTGAAGAGG					
tet B F	TTGGTTAGGGGCAAGTTTTG	*tet B*	659			
tet B R	GTAATGGGCCAATAACACCG					
Sul 1 F	F:GCG CGG CGT GGG CTA CCT	*sul 1*	350	65	94 °C for 5 min, 34 cycles, 25 s at 94 °C, annealing temperature for 45 s, and 50 s at 72 °C, and 5 min at 72 °C for the final extension.	
Sul 1 R	GATTTCCGCGACACCGAGACAA					
Sul 2 F	CGG CAT CGT CAA CAT AACC	*sul 2*	720	52	94° C for 5 min, 34 cycles, annealing temperature for 45 s, 25 s at 94 °C and 50 s at 72 °C, and 5 min at 72°C for the final extension.	
Sul 2 R	GTG TGC GGA TGA AGT CAG					

Table S4. List of oligonucleotide primer sequences and PCR conditions used for detection of genes associated with biofilm formation in *L. monocytogenes*.

Primers	Sequences (5′-3′)	Target Genes	Amplicon Size (bp)	Annealing T °C	PCR Conditions	References
luxS F	GGA AAT GCC AGC GCT ACA CTC TTT	*luxs*	208	58	94 °C for 2 min, 35 cycles, 30 s at 94 °C, annealing temperature for 30 s and 1 min, extension at 72 °C, and final extension of 7 min at 72 °C.	[15]
luxS R	ATT GCA TGC AGG AACTTC TGT CGC					
flaA F	GCG CAA GAA CGT TTA GCA TCT GGT	*flaA*	363			
flaA R	TTG AGT AGC AGC ACC TGT AGC AGT					

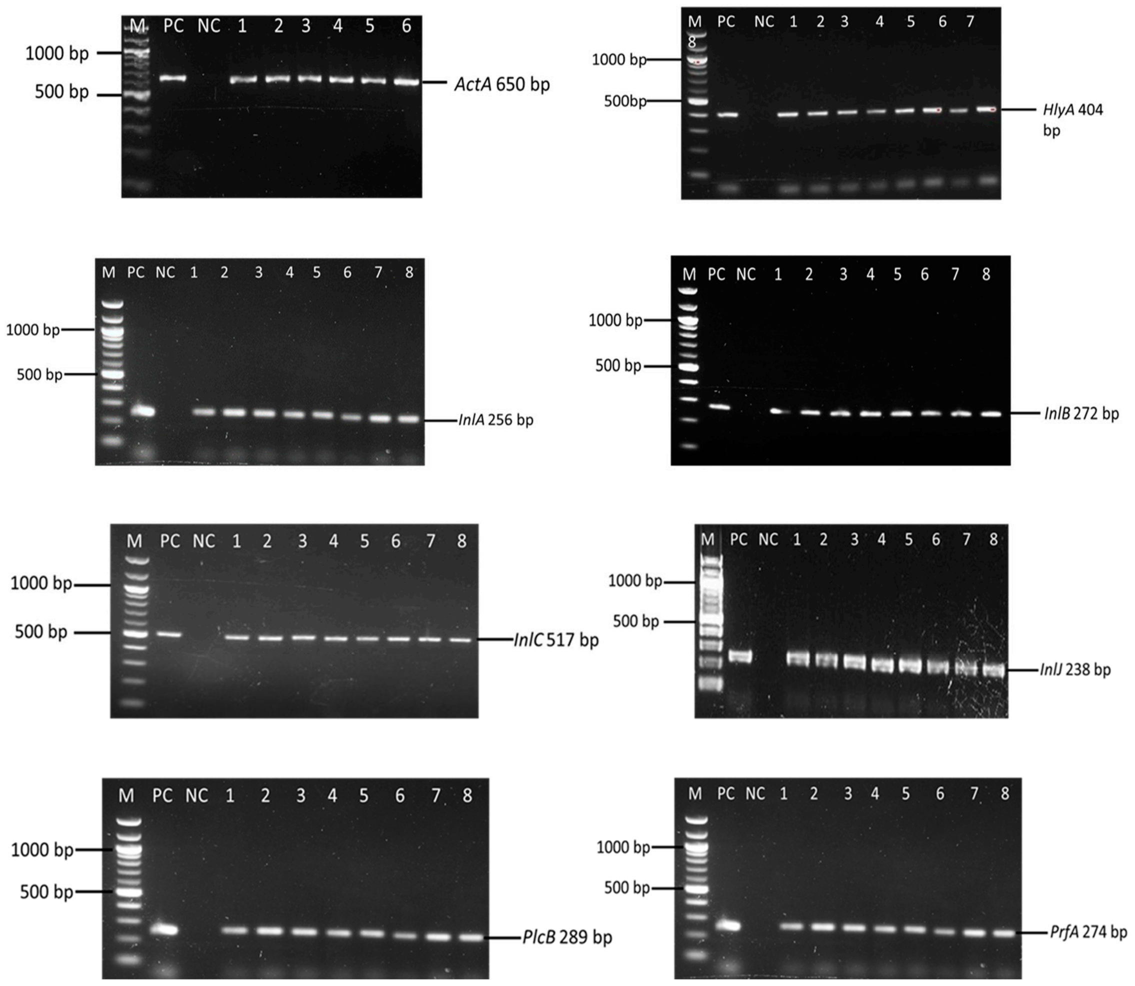

Figure S1. PCR amplification of virulence genes (M: Marker, PC: Positive control, NC: Negative control, 1-8: isolates amplification of virulence genes).

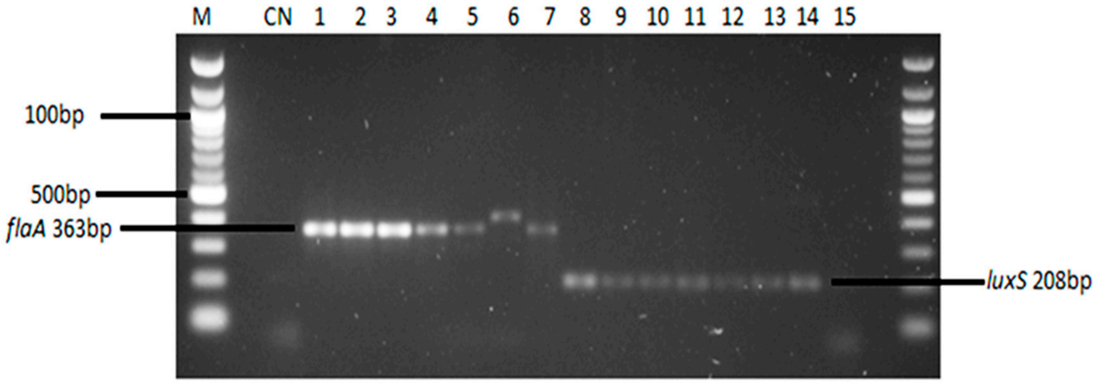

Figure S2. PCR amplification of biofilm genes (M: Marker, CN: Negative control, 1: Positive control, 2-7: isolates amplification of *flaA* gene, 8-13: isolates amplification of *LuxS* gene, 14: Positive control, 15: negative control).

References:

1. Bertrand, S.; Huys, G.; Yde, M.; D'Haene, K.; Tardy, F.; Vrints, M.; Swings, J.; Collard, J.-M. Detection and characterization of tet(M) in tetracycline-resistant Listeria strains from human and food-processing origins in Belgium and France. J. Med Microbiol. 2005, 54, 1151–1156. https://doi.org/10.1099/jmm.0.46142-0.

2. Mahm, M.M. Occurrence of Listeria monocytogenes in Raw Milk and Dairy Products in Noorabad, Iran. J. Anim. Veter- Adv. 2010, 9, 16–19. https://doi.org/10.3923/javaa.2010.16.19.

3. Ryu, J.; Park, S.H.; Yeom, Y.S.; Shrivastav, A.; Lee, S.-H.; Kim, Y.-R.; Kim, H.-Y. Simultaneous detection of Listeria species isolated from meat processed foods using multiplex PCR. Food Control 2013, 32, 659–664. https://doi.org/10.1016/j.foodcont.2013.01.048.

4. Coroneo, V.; Carraro, V.; Aissani, N.; Sanna, A.; Ruggeri, A.; Succa, S.; Meloni, B.; Pinna, A.; Sanna, C. Detection of Virulence Genes and Growth Potential inListeria monocytogenesStrains Isolated from Ricotta Salata Cheese. J. Food Sci. 2015, 81, M114–M120. https://doi.org/10.1111/1750-3841.13173.

5. Du, X.-J.; Zhang, X.; Wang, X.-Y.; Su, Y.; Li, P.; Wang, S. Isolation and characterization of Listeria monocytogenes in Chinese food obtained from the central area of China. Food Control 2016, 74, 9–16. https://doi.org/10.1016/j.foodcont.2016.11.024.

6. Liu, D.; Lawrence, M.L.; Austin, F.W.; Ainsworth, A.J. A multiplex PCR for species- and virulence-specific determination of Listeria monocytogenes. J. Microbiol. Methods 2007, 71, 133–140. https://doi.org/10.1016/j.mimet.2007.08.007.

7. Lomonaco, S.; Patti, R.; Knabel, S.J.; Civera, T. Detection of virulence-associated genes and epidemic clone markers in Listeria monocytogenes isolates from PDO Gorgonzola cheese. Int. J. Food Microbiol. 2012, 160, 76–79. https://doi.org/10.1016/j.ijfoodmicro.2012.09.011.

8. Kaur, S.; Malik, S.; Vaidya, V.; Barbuddhe, S. Listeria monocytogenes in spontaneous abortions in humans and its detection by multiplex PCR. J. Appl. Microbiol. 2007, 103, 1889–1896. https://doi.org/10.1111/j.1365-2672.2007.03414.x.

9. Egervärn, M.; Roos, S.; Lindmark, H. Identification and characterization of antibiotic resistance genes inLactobacillus reuteri-andLactobacillus plantarum. J. Appl. Microbiol. 2009, 107, 1658–1668. https://doi.org/10.1111/j.1365-2672.2009.04352.x.

10. Sutcliffe, J.; Grebe, T.; Tait-Kamradt, A.; Wondrack, L. Detection of erythromycin-resistant determinants by PCR. Antimicrob. Agents Chemother. 1996, 40, 2562–2566. https://doi.org/10.1128/aac.40.11.2562.

11. Delmani, F.; Jaran, A.S.; Tarazi, Y.A.; Masaadeh, H.; Zaki, O.; Irbid, T. Characterization of ampicillin resistant gene (blaTEM-1) isolated from E. coli in Northern Jordan. Asian J. Biomed. Pharm. Sci. 2017, 7, 11–15.

12. Grape, M.; Motakefi, A.; Pavuluri, S.; Kahlmeter, G. Standard and real-time multiplex PCR methods for detection of trime-thoprim resistance dfr genes in large collections of bacteria. Clin. Microbiol. Infect. 2007, 13, 1112–1118. https://doi.org/10.1111/j.1469-0691.2007.01807.x.

13. Bacci, C.; Boni, E.; Alpigiani, I.; Lanzoni, E.; Bonardi, S.; Brindani, F. Phenotypic and genotypic features of antibiotic resistance in Salmonella enterica isolated from chicken meat and chicken and quail carcasses. Int. J. Food Microbiol. 2012, 160, 16–23. https://doi.org/10.1016/j.ijfoodmicro.2012.09.014.
14. Poppe, C.; Martin, L.C.; Gyles, C.L.; Reid-Smith, R.; Boerlin, P.; McEwen, S.A.; Prescott, J.F.; Forward, K.R. Acquisition of Resistance to Extended-Spectrum Cephalosporins by Salmonella enterica subsp. enterica Serovar Newport and Escherichia coli in the Turkey Poult Intestinal Tract. Appl. Environ. Microbiol. 2005, 71, 1184–1192. https://doi.org/10.1128/aem.71.3.1184-1192.2005.
15. Warke, S.R.; Ingle, V.C.; Kurkure, N.V.; Tembhurne, P.A.; Prasad, M.; Chaudhari, S.P.; Barbuddhe, S.B. Biofilm Formation and Associated Genes in Listeria Monocytogenes. Indian J. Veter-Sci. Biotechnol. 2017, 12, 7–12. https://doi.org/10.21887/ijvsbt.v12i3.7079.
